# The effectiveness of an auditory temporal training program in children who present voiceless/voiced-based orthographic errors

**DOI:** 10.1371/journal.pone.0216782

**Published:** 2019-05-20

**Authors:** Mayra Monteiro Pires, Eliane Schochat

**Affiliations:** Department of Physical Therapy, Speech-Language Pathology and Occupational Therapy, School of Medicine, University of São Paulo, São Paulo, Brazil; Vita-Salute San Raffaele University, ITALY

## Abstract

**Introduction:**

Studies on children’s written production of the Brazilian Portuguese have shown that one of the most frequent phonological-based orthographic errors is the one related to voiceless/voiced phonemes. Children who make this type of error may have auditory temporal processing disorders, which can harm the perception of phonemes with similar characteristics.

**Aim:**

Verify the effectiveness of an auditory temporal training program based on activities adapted from the software Fast ForWord in the auditory temporal processing, i.e. the temporal ordering skill, and in reducing voiceless/voiced-based orthographic errors and the frequency of occurrence of these errors in the written production of the children.

**Method:**

Twenty-five children participated on this study. They were divided in two groups: experimental group consisting of 16 participants, who engaged in the auditory temporal training activities; and a placebo group consisting of nine participants, who engaged in passive visual activities. The behavioral measures applied in the pre-training evaluation, post-training and placebo evaluations were: i) auditory skill of temporal ordering by the Pitch Pattern Sequence Test; and ii) analysis of the amount of voiceless/voiced-based orthographic errors and the frequency of occurrence of these errors by the use of dictation.

**Results:**

No statistically significant differences were found concerning the placebo group in the pre-training and post-training evaluations, in all evaluation measures. However, statistically significant differences were found in the pre-training and post-training evaluations for the pitch pattern sequence test concerning the experimental group. These differences were specifically related to a reduction of the errors regarding fricative graphemes, and the frequency of occurrence of plosive and fricative graphemes.

**Conclusions:**

The auditory temporal training program was effective in improving the temporal ordering skill and reducing errors in the writing of children who made voiceless/voiced-based orthographic errors.

## Introduction

Learning a written language requires formal, constant and conscious learning based on the phoneme/grapheme association. During the orthographic acquisition, some processes occur until the formal alphabetic system is acquired according to the orthographic rules. Orthographic errors are commonly noticed all along these processes. However, the persistence of these errors is characterized as a spelling disorder [1]. Following dual-route models, in phonological spelling disorders, there is a sublexical procedure impairment that is related to regular transpositions from sound to letter, although lexical procedures are believed to contribute with the learning process of writing [2].

Concerning the development of writing abilities by children in transparent languages, phonological-based orthographic errors were found at all grades, which tend to decrease according to the years of schooling on an inversely proportional basis [3]. On the other hand, a study on Italian children’s writing with developmental dyslexia and dysorthographia, and normal development, demonstrated that children with developmental dysorthographia made more orthographical errors related to minimal acoustic distance substitutions in their writing, which have a tendency to disappear in the subsequential grades [4].

According to studies on children with normally developed writing of the Brazilian Portuguese, voiceless/voiced-based errors are the ones that occur more frequently. These studies demonstrate that the errors tend to decrease according to the years of schooling on an inversely proportional basis, similarly to the findings of the studies on Italian language learners previously mentioned [5,6]. These errors are related to the substitution of phonemes, which are distinguished by their corresponding pairs, voiceless or voiced, such as in /fan/ and /van/ or in /came/ and /game/ [7].

This error is believed to differ from other written language errors since it is possibly related to phonological system impairments, as a fragility of acoustic-to-phonological conversion [5] that may be associated to impediments concerning auditory differentiation of phonemes that are perceptually distinguished by one single temporal characteristic. Individuals who cannot properly distinguish specific traits of phonemes may have difficulty with the writing process that apparently are not only related to a specific level of the language, but also to a basic level of auditory differentiation [8].

It is believed that voiceless/voiced-based orthographic errors are related to auditory temporal processing impairments, which can be defined as the auditory perception of changes in the acoustic sign throughout time [9]. However, the origins of these errors are not very clear. It is believed that they are related to a primary sensory capacity to process fast sounds [10], or to a specific phonological encoding difficulty, in an abstract mental level [11].

Nowadays, behavioral auditory tests are used to evaluate auditory skills, such as the auditory temporal processing. One of these tests is the Pitch Pattern Sequence Test (PPST), which is an important tool to evaluate the auditory skill of temporal ordering. This skill is defined as the processing of multiple stimuli according to the order in which they occur in time, and is responsible for the individual’s correct differentiation and arrangement of the sounds they hear [12].

Behavioral tests applied to evaluate the auditory processing aims at measuring “bottom-up” aspects of sound processing in the central auditory nervous system, that is, the processing of physical mechanisms of auditory stimuli [13]. Studies have shown that “top-down” mechanisms, such as attention and memory, also contribute to behavioral tests that evaluate the auditory processing [14,15]. For example, memory and attention contribute 10% to the PPST [14]. A significant correlation between the PPST and the phonologic working memory was also reported [15].

In order to rehabilitate children with spelling disorders and auditory temporal processing disorders, the Scientific Learning organization conceived the Fast ForWord software (FFW). The main premise of this software is the stimulation of mechanisms that prompt the neural plasticity by using auditory activities focused on auditory temporal processing and linguistic aspects [16,17]. FFW activities use differentiation and ordering of frequencies according to the acoustic characteristics of the English language. The FFW linguistic stimuli are acoustically modified and present slow formant transition used to cope with difficulties related to auditory differentiation [16].

In Brazil, studies with dyslexic children adapted FFW’s activities in the language module. This adaptation used linguistic and non-linguistic stimuli based on characteristics of the Brazilian Portuguese (BP) consonants. Results indicated significant improvements in auditory temporal processing skills and language tests, such as phonological awareness and reading [18]. Improvements on the performance of auditory and linguistic abilities by the dyslexic children were associated to auditory learning, which can be defined as any improvement on the performance measured after an auditory activity and that lasted for a specific period of stimulation. Generally, this improvement can be transferred to auditory domains and other ones, such as cognition and language [19].

Auditory temporal training is believed to contribute to improvements on the auditory temporal ordering [20,21,22] and linguistic skills in individuals with oral and spelling disorders [23,24,25,26,27]. However, further studies should be conducted to evaluate the effectiveness of this type of intervention on specific orthographic errors that may be associated to auditory temporal processing impairments, since, according to the Rapid Temporal Processing Theory, children with spelling disorders may have difficulty processing fast sounds (i.e. consonant sounds) and may reproduce these perceptual difficulties in the written language [10].

Bearing the theoretical assumptions hitherto discussed, this paper aims at verifying the effectiveness of an auditory temporal training program (ATTP), based on activities adapted from the software FFW, in the auditory temporal processing, specifically, the temporal ordering skill, In this sense, this study aims at investigating whether there is a reduction in voiceless/voiced-based orthographic errors, as well as in the frequency of occurrence of these errors in the written performance of the children analyzed.

## Material and methods

This research was approved by the Research Ethics Committee of the *Hospital das Clínicas*, Medical School, University of São Paulo, under protocol number 315/15 and was funded by the *Fundação de Amparo à Pesquisa do Estado de São Paulo* (FAPESP). All parents signed a participation consent document on behalf of their children.

### Participants

The participants of this study were children with developmental dysorthographia who present voiceless/voiced-based orthographic errors in their writing and whose auditory temporal ordering skill was altered. Twenty-five children participated in the present study (15 boys and 10 girls). These children were 10–12 years old and were secondary students (fifth and sixth grades) of a public school situated in the University of Sao Paulo.

The children presented hearing thresholds up to 20dB with frequencies of 500–4.000 Hz, intellectual coefficient (IQ) from 90 to 109 points in the Wechsler Intelligence Scale for Children—III (WISC) test. The participants presented no neurological impairment, no speech sound disorders (evaluated with the picture naming of the ABFW test [28] and no ophthalmologic abnormalities.

In order to participate in this research, the children should not present reading disorders in the phonological-grapheme test [29]. In addition, 90% of their answers should be correct. During the evaluation, they were given a card with ten pseudowords and were supposed to identify the pseudoword that had been said by the evaluator. The phonological-grapheme test [29] aimed at verifying the acknowledgment of the phonological codification rules that are important to attest the proficiency in reading.

In addition, to select children with developmental dysorthographia, an evaluation of the writing consisting of a dictation of a with157 words-text was used as a screening test. The phonemes used were /p/, /b/, /t/, /d/, /k/, /g/ /f/, /v/, /s/, /z/, /ch/ and /j/, which were phonologycally balanced. The text dictated was proposed in a Brazilian research to select children with developmental dysorthographia as participants [30]. The selected children presented a minimum of 25% occurrences of voiceless/voiced-based orthographic errors in their writing [28].

The children were divided in two groups: a placebo group (PG) of nine children that underwent a placebo training, and an experimental group (EG) of 16 children that underwent the auditory temporal training program (ATTP). Children were randomly selected to participate in either the PG or EG.

The auditory processing test as well as the writing tests were applied in the pre-training, post-training and placebo evaluations to both groups to verify their performances after each specific type of intervention. Children from both groups were re-evaluated a month after each intervention process. The tests used are described as follows.

### Auditory temporal processing skill

PPST [31]: this test was applied in the GSI 61, Grason-Stadler audiometer, using 50dB above the tritonal average, in a Vibrasom acoustic cabin. It was used to evaluate the auditory temporal ordering skill.

This test consists of 30 trials with three tones of frequency, of which two are equal and one differs from the others concerning the frequency. This test presents sharp frequencies stimuli of 1122 Hz and bass frequencies of 880 Hz and duration of 150 ms. Inter-stimulus interval were of 2000 milliseconds. Within the sequence presented, children were advised to say “thin” whenever the stimulus heard was sharp and “thick” whenever a bass sound was heard. In the end of the test, the correct answers were counted; each item corresponded to 3,3, considering the number of trials (total of 30 hits). All points were recorded in the answer-sheet. Normality standard values of PPST consist of 70% of hits [32].

### Written language evaluation

Dictation list [30]: is composed by words with the phonemes /p/, /b/, /t/, /d/, /k/, /g/ /f/, /v/, /s/, /z/, /ch/ and /j/ in all syllabic positions, in stressed and unstressed syllables. The list was constructed by two-syllable words, three-syllable and polysyllable words, with the aforementioned graphemes phonologically balanced. This list of dictation was constructed in the Department of Phonology of the Linguistic Graduate Program at the Federal University of Santa Catarina, Brazil, and it was used in a research with 234 children.

Children were instructed to listen to the words and write them in a sheet of paper. The criteria for participation in the research was the minimum of 25% occurrences of voiceless/voiced-based orthographic errors, as it is commonly considered in the language evaluation [28]. The analysis was conducted according to the percentage of errors related to the amount of plosive and fricative graphemes errors in children’s writing and the frequency of occurrence, in order to answer the following question: how many times does the specific error appear in these graphemes.

### ATTP

Sessions of the ATTP were conducted twice a week, successively, totalizing eight sessions of 30 minutes that occurred in the computer room of the children’s school. Participants used Intel computers individually, provided with Philips headphones. The ATTP used four games with activities adapted from the FFW, which used stimuli based on characteristics of the BP. The games are described below.

- Monkey Game [33]
  This game was used to stimulate the auditory skill of differentiation and temporal ordering by using non-linguistic stimuli. The stimuli ranged between 60 and 20 milliseconds; inter-stimulus interval varied from 500 to 20 milliseconds (ms), whereas the frequency varied 16 octaves per second. Initial and final frequencies ranged between 0,5 kHz, 1kHz, 2 kHz and 4 kHz, with upward sounds (low frequencies to high frequencies) and downward sounds (high frequencies to low frequencies). In the first stage, the child was supposed to differentiate the stimuli; in the following stage, they had to put two stimuli in order, and having achieved the points necessary to go to the last stage, they were instructed to put three stimuli in order.
- Parrot Game [33]
  This game was used to stimulate the differentiation and ordering of linguistic syllable sounds with minimal distance pairs, with plosives and fricatives, by using speech stimuli acoustically modified, through the association of figures. Linguistic stimuli were acoustically expanded in time, in 100/ 80/ 60/ 40/ 20/ 15/ 10/ 0%, and were amplified in 10/ 8/ 6/ 4/ 2/0 dB, with inter-stimulus interval of 500/ 400/ 300/ 200/ 170/ 140/ 110/ 90/ 60/ 30/ 10 ms. Children were supposed to listen to a pair of syllables and associate it to the corresponding figures in the order they were presented to them.
- Memory Game [34]
  This game was used to stimulate the phoneme-grapheme association with speech stimuli acoustically modified by the auditory and visual memories. Compound words consonants of minimal distance pairs with fricatives and plosives were expanded in 60/30/0% and amplified in 7/5/0 dB to highlight acoustic differences between words and false-words with a card game. Children were supposed to find the corresponding pairs.
- Change Listen Game [34]
  This game was used to stimulate the phoneme-grapheme association by using speech stimuli acoustically modified, associated to letters of different visual speeds, which varied from 4, 5 seconds to 700 ms. Here, the principles of acoustic expansion in time and amplification were the same used in the Memory Game and in the consonants of words and false-words of minimal distance pairs with fricatives and plosives. As the expansion of speech stimuli of consonants was reduced, the visual speed in which the stimuli were presented on the computer screen was increased. The child was supposed to click on a specific button every time a word or a false-word different from the previous one was presented.

### Placebo training (PT)

A passive visual activity was applied as a placebo training. The child was supposed to watch eight episodes of a 30-minute TV-series at home under the supervision of an adult. The children watched two episodes per week, during one month, and by the end of each episode, they had to hand in a review. This type of PT had been used in other research [35] and it became a viable option for using in this study.

The results of the PT and the ATTP in the experimental group and the placebo group underwent a statistical analysis at the moment of evaluation before (pre-training) and after (post-training) the auditory and the PT in the measures of the PPST and the dictation taken by the children. Wilcoxon non-parametric statistical test was applied in order to compare the averages of the evaluations at pre-training and post-training moments (intra-group analysis) of both EG and PG. A significance level of 0,05 (5%) was used in this study. In general, values considered statistically significant are marked with one asterisk (*) when equal to or less than 0,05; with two asterisks (**) when equal to or less than 0,01; and marked with three asterisks (***) when equal to or less than 0,001. The Mann-whitney test with significance level of 0,05 (5%) was used to verify if there were differences between the EG and the PG before applying the two types of training.

## Results

### Verification of the groups’ performance before the two types of training

Table 1 informs the average amount of correct answers (in percentage terms) regarding the PPST.It also informs the ortographic erros the plosive and the fricative phonemes, frequency of occurrence of plosive (FOP) and frequency of occurrence of fricatives (FOF) phonemes, as well as the standard deviation, minimum, maximum and p-value for each variable in the pre-training evaluations of both EG and PG according to the Mann-whitney test.

**Table 1:** Comparison between the variables before each type of training.

Var.	Evaluation	N	Mean	Standard deviation	Min.	Max.	p-value
**PPST**	Pre-training (EG)	9	60,00	10,31	45,00	80,00	**0,931**
	Pre-training (PG)	16	56,44	11,42	30,00	75,00	
**Plosiv.**	Pre-training (PG)	9	0,67	0,71	0,00	2,00	**0,863**
	Pre-training (EG)	16	0,56	0,81	0,00	3,00	
**Frica.**	Pre-training						
	(PG)	9	0,89	0,60	0,00	2,00	**0,796**
	Pre-training						
	(EG)	16	1,00	0,89	0,00	3,00	
**FOP**	Pre-training						
	(PG)	9	22,00%	21,30	0,00	44,00	**0,931**
	Pre-training (EG)	16	17,19%	20,86	0,00	55,00	
**FOF**	Pre-training						
	(PG)	9	31,78%	19,40	0,00	55,00	**0,436**
	Pre-training (EG)	16	33,00	18,41	0,00	55,00	

Note: Var.: variable, Plosiv: plosives, Frica: fricatives. PG: placebo group; EG: experimental group; N: number of participants, FOP: frequency of occurrence of plosive; FOF: frequency of occurrence of fricatives.

Table 1 shows that statistically significant were not found when comparing the groups in the pre-training evaluation of the PG and the EG regarding the following variables: PPST (p = 0,931), plosives (p = 0,863) and fricative (p = 0,796), FOP (0,931) and FOF (0,436).

### Effect of the ATTP and PT for behavioral evaluation of the auditory processing

Table 2 informs the average amount of correct answers (in percentage terms), standard deviation, minimum, maximum and p-value (Wilcoxon) of the PPST regarding the EG and the PG evaluations.

**Table 2:** Percentage of correct answers in the PPST per group analyzed.

Group	Evaluation	N	Mean	Standard deviation	Min.	Max.	p-value
**PG**	Pre-training	9	60,00	10,31	45,00	80,00	**0,23**
	Post-training	9	56,11	11,40	30,00	65,00	
**EG**	Pre-training	16	56,44	11,42	30,00	75,00	0,001***
	Post-training	16	77,81	13,29	55,00	100,00	

Note: PG: placebo group; EG: experimental group; N: number of participants

*** statistically significant equal or less than 0,001.

Table 2 shows differences in the average amount of correct answers concerning the pre-training evaluation by the PPST (56,44%) and the post-training evaluation of the EG (77,81%). These results show a more relevant percentage of correct answers for this group after the execution of the ATTP. According to the Wilcoxon test, the auditory temporal ordering skill results of the EG show statistically significant (p = 0,001) differences between the two evaluations regarding the frequencies and the interhemispheric transfer.

As for the PG, Table 2 indicates that the average of correct answers in the PPST was worse in the post-training evaluation (56,11%) than in the pre-training evaluation (60,00%). According to the Wilcoxon test, differences between the two evaluations were not statistically significant (p = 0,23) for this group.

### Effects of the PTAT and the PT in the results related to writing

Table 3 informs the average values of correct answers, standard deviation, minimum, maximum and p-value (Wilcoxon) regarding the amount of errors in plosive and fricative graphemes (in percentage terms), concerning the two evaluations undertaken by the experimental and the placebo group.

**Table 3:** Amount of errors related to plosive and fricative graphemes in the two groups, considering the two evaluations.

Variable	G	Evaluation	N	Mean	Standard deviation	Min.	Max.	p-value
**Plosives**	PG	Pre-training	9	0,67	0,71	0,00	2,00	0,66
		Post- training	9	0,56	1,01	0,00	3,00	
	EG	Pre-training	16	0,56	0,81	0,00	3,00	0,53
		Post- training	16	0,44	0,63	0,00	2,00	
**Fricatives**	PG	Pre-training	9	0,89	0,60	0,00	2,00	0,32
		Post- training	9	0,78	0,67	0,00	2,00	
	EG	Pre-training	16	1,00	0,89	0,00	3,00	0,005**
		Post- training	16	0,25	0,45	0,00	1,00	

Note: PG: placebo group; EG: experimental group; N: number of participants, o group; EG: experimental group; N: number of participants

** statistically significant equal to than or less than 0,01.

Table 3 shows a reduction of voiceless/voiced-based orthographic errors in plosive graphemes when comparing pre-training (0,56%) and post-training evaluations of the EG. However, according to the Wilcoxon test, no statistically significant differences were found between the two evaluations (p = 0,53). The same results were found for the PG, since no statistically significant differences were found between the two evaluations (p = 0,66) when comparing the average amount of voiceless/voiced-based orthographic errors in plosive graphemes in the pre-training (0,67%) and post-training (0,56%) evaluations.

As for the voiceless/voiced-based orthographic errors in fricative graphemes in the EG, Table 3 shows that there was a reduction in these processes when comparing the average of these errors in the pre-training (1,00) and the post-training (0,25) evaluations of the EG. According to the Wilcoxon test, statistically significant differences were found between the two evaluations of this group (p = 0,005). In the PG, a reduction of the voiceless/voiced-based orthographic errors in fricative graphemes was found when comparing the average of these errors in the pre-training (0,89%) and post-training (0,78%) evaluations. However, differences between the two evaluations in the PG were not statistically significant (p = 0,32).

Table 4 informs the EG and the PG’s average of correct answers, standard deviation, minimum, maximum and p-value (Wilcoxon) regarding the FOP and FOF of the two evaluations in percentage terms.

**Table 4:** Frequency of occurrence of plosives and frequency of occurrence of fricatives in both groups, in all evaluations.

Variable	G	Evaluation	N	Mean	Standard deviation	Min.	Max.	p-value
**FOP**	PG	Pre-training	9	22,00%	21,30	0,00	44,00	**0,34**
		Post-training	9	14,67%	22,68	0,00	55,00	
	EG	Pre-training	16	17,19%	20,86	0,00	55,00	0,04*
		Post-training	16	6,88%	9,74	0,00	22,00	
**FOF**	PG	Pre-training	9	31,78%	19,40	0,00	55,00	**0,20**
		Post-training	9	24,44%	18,88	0,00	44,00	
	EG	Pre-training	16	33,00%	18,41	0,00	55,00	0,001***
		Post-training	16	4,13%	7,91	0,00	22,00	

Note: PG: placebo group; EG: experimental group; N: number of participants; FOP: frequency of occurrence of plosive; FOF: frequency of occurrence of fricatives

* statistically significant equal or less than 0,05

*** statistically significant equal or less than 0,001.

Table 4 shows the EG’s frequency of occurrence of orthographic errors in voiced/voiceless plosive graphemes. The reduction of these processes was observed in the pre-training (17, 19%) and in the post-training evaluation (6,88%), with statistical differences (p = 0,001), according to the Wilcoxon test. PG’s pre-training results show an amount of 31,78%. A reduction was observed in the post-training results (24,44%), with no statistical differences (p = 0,20).

As for the EG’s frequency of occurrence of orthographic errors in voiced/voiceless fricative graphemes, Table 3 shows a reduction of these processes when comparing the average of the frequency of occurrence of such errors in the pre-training (33,00%) and the post-training (4,13%) evaluations. According to the Wilcoxon test, statistically significant differences (p = 0,001) were found between the two evaluations of this group. A reduction in the frequency of occurrence of the same error was observed in the PG when comparing the average of pre-training (31,78%) and post-training (24,44%) evaluations. However, these differences were not statistically significant (p = 0,20).

## Discussion

A PT was used in this study in order to monitor the results. It was supposed to point out whether the findings, after the ATTP, were a result of the proposed intervention, a test-retest effect, or a consequence of the maturing process of the children. The test-retest effect is related to a better performance in behavioral tests due to its double application, which can suggest that the test was “learned” by the participant [36]. On the other hand, the maturing process is related to the child exposure to external stimuli that can improve their cognitive and auditory skills [37].

Table 1 shows that there were not statistically significant differences between the group’s performance regarding the variables used in this study in the EG’s and the PG’s pre-training evaluations. These results suggest that the groups presented similar performances to that of the PPST and the evaluation of the writing before each type of training. Therefore, it is believed that the results that will be discussed afterwards in the present study show the effectivity of the ATTP.

Tables 2, 3 and 4 show that no statistically significant differences were found between the results of the PG’s pre-training and post-training evaluations concerning the PPST and the measures of the writing evaluation. Studies describe the use of a PT in order to evaluate the effectiveness of the auditory training on behavioral measures in the auditory processing and language. Generally, the results from participants that underwent the PT do not meet the results from participants who underwent interventions, for there was no improvement on the performance regarding these measures when the participants were re-evaluated [38,39,40].

Since in this study no statistically significant differences were found concerning all the behavioral tests applied to the PG, the results described hereafter were attributed to the effectiveness of the ATTP in the EG.

### Effect of the ATTP on the behavioral measures of the auditory processing

Table 2 shows that the EG’s average of correct answers was lower than the pre-training evaluation normality standards (M = 56,44%) of the PPST ^(31)^, which is of 70% hits. Regarding the post-training evaluation, the average of correct answers in the PPST improved (M = 77,81%), expressing a statistically significant difference (p = 0,001). The group’s post-training evaluation results met the normality standards. These results suggest that the ATTP was effective in improving the EG-children’s performance regarding the auditory ordering skill and the inter-hemispheric transfer.

The ATTP used the Monkey Game, which presents a scale of difficulty regarding differentiation and ordering of frequencies. EG’s results on Table 2 demonstrate that, besides improving the perceptual auditory temporal ordering ability, the auditory learning was transferred from the Monkey Game’s activities to the PPST, which presented a maximum of three tones of frequency that should be ordered in a specific period of time. The improvement on the performance of the auditory temporal ordering skill after the use of the ATTP meets research findings [20,21,22] that demonstrate improvement on the performance of this auditory skill in children who have learning and language disorders. Consequently, an improvement on the sensorial mechanism (“bottom-up”) was observed regarding differentiation and ordering of frequencies due to the neural plasticity in the central auditory nervous system, driven by the learning process [41,42].

From a “top-down” perspective, the EG’s improvement on the performance of the PPST can be justified by the fact that the activities of the ATTP encompassed symbolic association of visual and auditory stimuli. In this study, the PPST was applied in the verbal stage (inter-hemispheric transfer), that is, children were supposed to say whether the stimuli they heard was “thick” or “thin”.

Therefore, since the ATTP activities use a symbolic association of sounds and linguistic and non-linguistic symbols, EG-children may have attributed a more accurate meaning to bass and sharp sounds, which must have contributed to a better performance in the verbal stage of the PPST, and facilitated the interhemispheric transfer. Children with language disorders may have impairments in the phonological representation responsible for associating letters (symbols) to their respective sounds [43]. The stimulation provided by ATTP’s activities may have helped children to overcome these impairments and influenced the results of the PPST in the post-training evaluation.

Studies argue that PPST is a test that not only evaluates the auditory temporal ordering skill, but also includes “top-down” contributions. This is because the participant must put in order the tones heard in a given period of time, thus, it requires the use of the memory ability and other superior executive functions, such as attention, so they can properly respond to the test [14,15].

ATTP activities involved both “bottom-up” and “top-down” aspects on the stimuli used and types of activities. For instance, the Monkey Game used non-linguistic stimuli, whereas the Parrot Game used linguistic stimuli, in which children were supposed to guess the order in which the stimuli was presented, with different levels of inter-stimulus interval.

It is possible to infer that attention and memory were indirectly stimulated, since studies posited that auditory training can drive the generalization process from auditory learning to cognitive domains, such as attention and memory [19,44,45]. Therefore, the improvement on these domains may have influenced the “top-down” contribution of the PPST, providing better results in the EG’s post-training evaluation.

### Effect of the ATTP in the results related to writing

Table 3 demonstrates a reduction in the EG’s average percentage of voiceless/voiced-based orthographic errors concerning the plosive graphemes in the post-training evaluation. However, no statistically significant differences were found regarding the reduction of these errors when comparing pre-training and post-training evaluations. The following conclusions about the Table 3 findings were based on significantly low percentage distributed in the sample of both groups that need to be analyzed in a larger sample so that consistent results can be achieved. However, it is important to describe them in the present study since this is an initial experimental research.

As for the fricative graphemes, statistically significant differences regarding the reduction of the average percentage were found in the EG’s pre-training and post-training evaluations. Although the percentages in Table 3 are very low, it is believed that the results produced are due to the effectiveness of the ATTP in helping the children of the EG to reduce orthographic errors that are related to fricative graphemes.

Apparently, non-significant results for orthographic errors related to plosive graphemes (Table 3) might be associated to the limitations of the acoustic expansion in time of linguistic stimuli that produce plosive phonemes. These phonemes are produced by a constant obstruction of the airflow at a specific point of the vocal tract area, followed by an explosion and a fast liberation of this airflow [46]. Due to the phono-articulatory characteristics of plosive phonemes, which present obstruction followed by a fast explosion, the acoustic expansion may not have facilitated the perception of differences between voiceless and voiced pairs. In this sense, EG-children could not perceive through auditory means the distinction between the two similar phonemes; thus, the association to the grapheme was not effective by the time the post-training evaluation was conducted.

As for the temporal measures, plosive phonemes showed lower duration values (31 to 63,75 ms), whereas fricative phonemes showed longer duration values (96,25 to 149,85 ms) when compared to plosive phonemes [47]. The statistically significant difference in the reduction of fricative errors concerning the pre-training and the post-training evaluations of the EG-children, according to the Table 3 (p = 0,005), are believed to occur due to the expansion of the linguistic stimuli that facilitated the perception of the existing differences between voiceless and voiced pairs of fricative phonemes because of their long duration. As a result, children may have noticed the contrast between voiceless and voiced fricatives.

Other hypothesis to the EG-children’s reduction of orthographic errors related to fricative graphemes is the phono-articulatory characteristic of the fricative phonemes, since they do not involve total obstruction of the airflow, that is, they are continuous [46]. In this sense, it is believed that the acoustic expansion in time of these phonemes during the activities of the ATTP may have turned the acoustic differences between voiced and voiceless sounds into more perceptible sounds. This possibly facilitated the correct phoneme-grapheme association of the fricative graphemes.

Statistically significant differences had not been found regarding the reduction in the percentage of voiceless/voiced-based orthographic errors in plosive graphemes in the EG’s post-training evaluation. As Table 3 shows, there was more reduction in the frequency of occurrence of errors related to these graphemes according to the results of the average performance of the pre-training and the post-training evaluations of the EG. Based on these results, it is possible to argue that orthographic errors were still being found in the children’s writing during the post-training evaluation, though the amount of these errors was lower than the amount found in the pre-training evaluation.

With these results, it is possible to infer that EG-children were under the correct learning process of phoneme-grapheme association regarding the plosive graphemes, and that these orthographic errors can be unsystematic, that is, the process of phonological representation, which determines the correct association of plosive phonemes to plosive graphemes, is still not entirely fulfilled [48]. In this sense, it is suggested that if the EG-children undergo ATTP sessions for a longer period, the learning from fricative graphemes will be transferred to plosive graphemes.

Table 4 also displays a reduction in the percentage of orthographic errors related to fricative graphemes when comparing pre-training and post-training evaluations (p = 0,001), with higher statistical significance than the frequency of occurrence of errors related to plosive graphemes. These results indicate a reduction in the amount of times the errors related to fricative graphemes were found in the EG-children.

Apparently, the improvement on the children’s performance regarding the fricative graphemes was influenced by the acoustic expansion in time of linguistic stimuli provided by the ATTP activities. These results corroborate studies that found speech stimuli acoustically expanded in time through FFW-based auditory training to be more effective in providing benefits in language tests than auditory training based on linguistic stimuli that are not acoustically expanded in time. Results from these studies demonstrated that children who underwent auditory training with speech acoustically expanded in time showed more significant improvement in linguistic tests than the children who had undergone auditory training based on linguistic stimuli without acoustic expansion in time [16,49].

It is possible to argue that the speech stimuli acoustically expanded in time provided acoustic perception that had not been noticed previously in the EG-children. Therefore, the changes in the central auditory nervous system through neural plasticity made possible the modification of phonological representations, which may have facilitated the phoneme-grapheme association, similarly to the children’s post-training evaluation results.

Perception of temporal characteristics of non-linguistic stimuli used in the Parrot Game are believed to be the reason of the EG’s reduction of voiceless/voiced-based orthographic errors regarding fricative graphemes (Table 3) and of the frequency of occurrence of these errors regarding plosive and fricative graphemes (Table 4). Before the game was conceived, a study had been conducted to verify the acoustic parameters of the BP plosive and fricative consonants regarding duration, frequency, and variation of octaves per second [47]. It is believed that the differentiation of frequency, intensity and duration of these stimuli are of utmost importance to the processing of complex stimuli, such as phonemes. Studies that used non-linguistic auditory stimuli in the auditory training indicated an improvement on the performance of linguistic reading abilities related to an effective grapheme-phoneme association [25,50,51]. These studies corroborate the hypothesis that the Monkey Game’s activities may have also provided better results in the EG-children’s writing.

As the ATTP activities made use of both linguistic and non-linguistic auditory stimuli associated to visual stimuli, it is possible to infer that the association mechanism of auditory stimuli and visual stimuli contributed to an effective grapheme-phoneme association. This is because auditory training based on auditory and visual stimuli provides better perceptual representation of voiced and voiceless phonemes in auditory pathways [51].

Concerning the limitations of this study, it is important to stress that different types of training were applied. The ATTP was used in the EG, whereas the PT was used in the PG. The ATTP was applied in the school where the reseach took place, in the presence of the researcher, to control the proposed activities. On the other hand, the PT was applied at the children’s home. Although the PG-children had done a writing activity based on a TV series to attest that they did the activity, ideally these activities should have been performed in the school, in the presence of the researchers, in order to control the study’s environment. For example, the duration of the PT and the concentration of the PG-children could be controlled by the researcher during the training.

Studies that compare different techniques of auditory training need a similar environmental control of both placebo and experimental groups. It is believed that the difference on the tests application, that is, the use of two different environments (school vs. home) may have affected the results of the final re-evaluation of the placebo group, since the experimental group had its performance on the ATTP’s activities controlled by the presence of the researchers.

## Conclusions

The results of this study show that the ATTP was effective in improving the auditory temporal ordering skill in children. As for the writing language evaluation, after the use of the ATTP, there was a reduction of voiceless/voiced-based orthographic errors regarding fricative graphemes, and a reduction in the frequency of occurrence of errors regarding plosive and fricative graphemes. Therefore, it is possbible to argue that the ATTP was effective in improving processes related to voiceless/voiced-based orthographic errors and to the auditory temporal processing in the population analyzed.

## Supporting information

S1 Table This is the S1 Table with PPST, plosives and fricatives errors, FOP and FOF scores. (XLSX)
